# Effects of Early Initiation of High-Dose Dexamethasone Therapy on Pro-Inflammatory Cytokines and Mortality in LPS-Challenged Mice

**DOI:** 10.3390/healthcare10071247

**Published:** 2022-07-04

**Authors:** Ji-young Son, Won Gun Kwack, Eun Kyoung Chung, Sooyoung Shin, Yeo Jin Choi

**Affiliations:** 1 Department of Clinical Pharmacy, Graduate School of Pharmacy, CHA University, Seongnam 13488, Korea; a01077218649@gmail.com; 2 Division of Pulmonary, Allergy and Critical Care Medicine, Kyung Hee University Hospital, Seoul 02447, Korea; wongunnim@naver.com; 3 Department of Pharmacy, College of Pharmacy, Kyung Hee University, Seoul 02447, Korea; 4 Department of Regulatory Science, Graduate School, Kyung Hee University, Seoul 02447, Korea; 5 Department of Pharmacy, Kyung Hee University Hospital at Gangdong, Seoul 05278, Korea; 6 Department of Clinical Pharmacy, College of Pharmacy, Ajou University, Suwon 16499, Korea; 7 Research Institute of Pharmaceutical Science and Technology (RIPST), Ajou University, Suwon 16499, Korea

**Keywords:** corticosteroid, dexamethasone, lipopolysaccharide, acute inflammation, sepsis

## Abstract

This study aims to explore the effects of early dexamethasone therapy at low to high doses on the survival and inflammatory responses in lipopolysaccharide (LPS)-challenged mice. We performed two-series experiments to explore the impact of early dexamethasone therapy at different doses (0.5 mg/kg, 1.5 mg/kg, and 5 mg/kg; PO) on pro-inflammatory cytokine levels, including tumor necrosis factor-alpha (TNF-α) and interleukin-6 (IL-6), as well as survival in LPS-treated mice (10 mg/kg, IP). Dexamethasone was administered daily from 24 h before and 5 days after LPS challenge. Dose-dependent improved survival was demonstrated with dexamethasone (*p* < 0.05). Body weight was significantly decreased within 24 h of LPS injection, with significantly greater weight loss in the dexamethasone groups (*p* < 0.05). Weight changes were significantly associated with the days after LPS administration (*p* < 0.01), but not with the dexamethasone dose (*p* > 0.05). Mice treated with high-dose dexamethasone (5 mg/kg) had a significantly lowered serum TNF-α (134.41 ± 15.83 vs. 408.83 ± 18.32) and IL-6 (22.08 ± 4.34 vs. 91.27 ± 8.56) compared with those without dexamethasone. This study provides essential insights that the suppression of early-phase hyperactivation of pro-inflammatory activities through the early initiation of high-dose dexamethasone therapy increases sepsis-related prognosis.

## 1. Introduction

Sepsis is an acute, life-threatening condition involving systemic inflammatory responses to microbial infections mediated by innate immune and vascular system activations, subsequently inducing severe organ dysfunctions or septic shock [1]. Because of the increase in the number of immunocompromised patients and in the elderly population, the incidence of sepsis is steadily increasing each year [2]. Sepsis is a prominent cause of in-patient mortality accounting for 30% to 50% of deaths, and approximately 11.0 million sepsis-related fatalities were reported in 2017 [3]. Early initiation of appropriate management is warranted in order to improve treatment outcomes in patients with sepsis [4]. According to the guidelines for the management of sepsis, published by the Society of Critical Care Medicine (SCCM) and the European Society of Intensive Care Medicine (ESICM), early antibiotic administration within an hour of sepsis diagnoses and immediate fluid resuscitation are recommended in order to maximize survival in these patient populations [4]. The Surviving Sepsis Campaign Guidelines updated in 2021 suggested the use of intravenous corticosteroids for adults with septic shock and proper vasopressor therapy in order to accelerate the resolution of shock [4].

In clinical practice, various corticosteroids are available with differences in the overall pharmacological potency as well as relative glucocorticoid anti-inflammatory to mineralocorticoid activity [5,6]. The optimal agent, dose, timing of initiation, and duration of corticosteroid therapy in patients with sepsis remain largely uncertain. The latest sepsis guidelines suggest low-dose (<400 mg daily, typically 200–300 mg per day) hydrocortisone for patients with septic shock accompanied by adrenal insufficiency or refractory hypotension despite vasopressor therapy and adequate fluid resuscitation [4]. Hydrocortisone is a unique corticosteroid because of its relatively high mineralocorticoid activity (glucocorticoid-to-mineralocorticoid activity ratio = 1:1), making it a preferred agent in patients with septic shock and persistent vasopressor requirements [4,7]. However, conflicting evidence has been published regarding the role of high-dose corticosteroid (equivalent to ≥400 mg hydrocortisone daily) or alternative corticosteroids with a potent glucocorticoid anti-inflammatory activity [8,9]. According to previous guidelines for corticosteroid insufficiency associated with critical illness such as septic shock, high-dose corticosteroids or dexamethasone (DEX), which has the greatest glucocorticoid potency but minimal to no mineralocorticoid activity, are not recommended for septic shock because3 of their immediate, potent, and prolonged suppression of the hypothalamic−pituitary−adrenal (HPA) axis [7,10]. The concern is that high-dose corticosteroids or DEX potentially increase the risk of steroid-related complications, including myopathy and superinfections. However, our recent meta-analysis with randomized clinical trials demonstrated improved survival outcomes in patients with sepsis receiving DEX therapy at different doses. With the increasing use of DEX for septic patients with coronavirus disease 2019 (COVID-19) requiring hospitalization for external oxygen supply, invasive mechanical ventilation, or extracorporeal membrane oxygenation [11,12], the therapeutic benefits of DEX in sepsis should be re-evaluated to optimize treatment strategies.

The most current Surviving Sepsis Campaign Guidelines in 2021 suggest initiating intravenous low-dose hydrocortisone in vasopressor-dependent patients after at least 4 h of vasopressor therapy in order to maintain the target blood pressure. However, based on recent clinical studies, the early initiation of corticosteroid therapy immediately after recognizing sepsis improved treatment outcomes, including mortality, in the intensive care unit (ICU) in patients with septic shock [13,14]. Considering the retrospective observational study design and the small number of patients included in these recent studies, robust evidence from large-scale, well-designed clinical studies is warranted to determine the therapeutic roles of early corticosteroid therapy with a high glucocorticoid activity in septic shock. It may be extremely challenging to perform clinical studies assessing the safety and effectiveness of early DEX therapy at low to high doses in patients with septic shock considering the potentially harmful effects and extensive requirement of time and labor to recognize sepsis immediately [15]. Therefore, we performed a preclinical study to explore the effects of early DEX therapy at low to high doses on survival and inflammatory responses in lipopolysaccharide (LPS)-challenged mice. Although the findings of our current study may not directly assist in designing and conducting future clinical studies to confirm the therapeutic benefits of high-dose DEX in septic shock as the mouse model does not exactly reproduce the pathophysiologic processes of sepsis in humans [16,17], these findings may support the importance of the early inhibition of pro-inflammatory activity in improving sepsis-related mortality, as well as prognosis in sepsis patients.

## 2. Materials and Methods

### 2.1. Materials and Animals

LPS was obtained from Sigma Aldrich (St. Louis, MO, USA) and DEX was obtained from Jeil Pharmaceuticals (Daejeon, Korea). Both LPS and DEX were dissolved in 0.9% sodium chloride (normal saline). Male C57BL/6N mice (6 weeks old, 18–23 g) were purchased from Orient Bio Co., Ltd. (Seongnam, Korea). All of the animal experiments were conducted in compliance with the Bioethical Standards for Animal Studies and the guidelines established by the Animal Care Committee of Chemon (Yongin, Korea). The experimental protocol was approved by the Experimental Animal Steering Committee of Chemon (approval number: 2020-06-008). The mice were acclimated to controlled conditions of temperature (23 ± 3 °C), humidity (55 ± 15%), and ventilation frequency (10–20 times/h) in the animal rooms of the Gyeonggi Bio Center with 12 h light/dark cycles.

### 2.2. Experimental Designs

#### 2.2.1. Survival of LPS-Challenged Mice

The mice were randomly assigned to the following four groups (N = 8 per group) for the survival analysis: (1) LPS only (intraperitoneal [IP] LPS 10 mg/kg), (2) LPS with DEX 0.5 mg/kg (LPS 10 mg/kg IP with oral [PO] DEX 0.5 mg/kg, equivalent to 52 mg hydrocortisone in human), (3) LPS with DEX 1.5 mg/kg (LPS 10 mg/kg IP with DEX 1.5 mg/kg PO, equivalent to 156 mg hydrocortisone in human), and (4) LPS with DEX 5 mg/kg (LPS 10 mg/kg IP with DEX 5 mg/kg PO, equivalent to 500 mg hydrocortisone in human) [16]. Dexamethasone was administered 24 h before LPS injection (first dose, day 0), 30 min before LPS injection (second dose, day 1), and then once daily from days 2 to 6. All of the mice under study were monitored once daily for body weights and survival throughout the experimental period.

#### 2.2.2. Cytokine Analysis of LPS-Challenged Mice

The mice were randomized into the following three groups (N = 6 per group): (1) normal control (NC), (2) LPS only (10 mg/kg IP), and (3) LPS with high-dose DEX (LPS 10 mg/kg IP with DEX 5 mg/kg PO). Dexamethasone was administered 24 h and 30 min before the LPS injection, and a single blood sample was collected from the posterior vena cava of each mouse with a 25-gauge needle 4 h after LPS administration. After the blood was allowed to coagulate, the collected blood samples were centrifuged, and the serum samples were stored frozen at −70 °C until cytokine quantitation. For the cytokine analysis, the serum concentrations of tumor necrosis factor-alpha (TNF-α) and interleukin-6 (IL-6) were quantified using an enzyme-linked immunosorbent assay (ELISA) kits (R & D Systems, Minneapolis, MN, USA).

### 2.3. Statistical Analysis

The survival of LPS-challenged mice was assessed based on Kaplan−Meier survival estimates, and the differences in survival probabilities among the four groups were statistically compared using the log rank test. The effect of DEX at varying doses (no DEX, 0.5 mg/kg, 1.5 mg/kg, and 5 mg/kg) on the body weights of mice over time was evaluated using linear mixed-effect modeling. Each individual animal was included as a random-effect variable; time (in days) and DEX dose were assessed as fixed-effect variables. The serum cytokine levels were comparatively analyzed among the tested animal groups using one-way ANOVA followed by the Newman−Keuls multiple range test. All of the statistical analyses were performed in R package (R version 4.0.3). Statistical significance was defined as *p*-values < 0.05.

## 3. Results

### 3.1. Survival and Weight Changes with Dexamethasone Treatment

**Figure 1:**
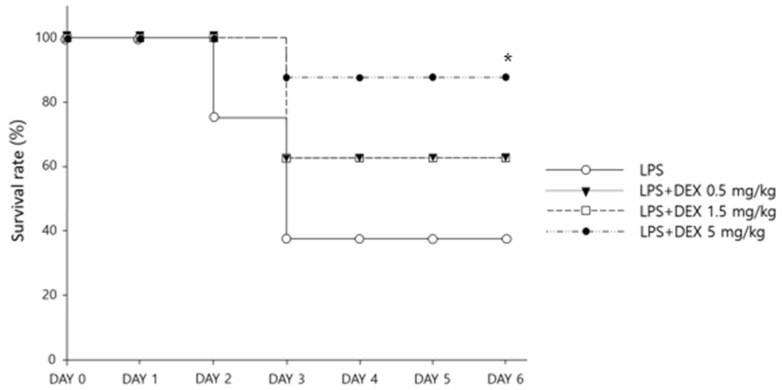
Survival rates of LPS-challenged mice based on the Kaplan−Meier estimates (N = 8 per group) (* *p* < 0.05).

**Figure 2:**
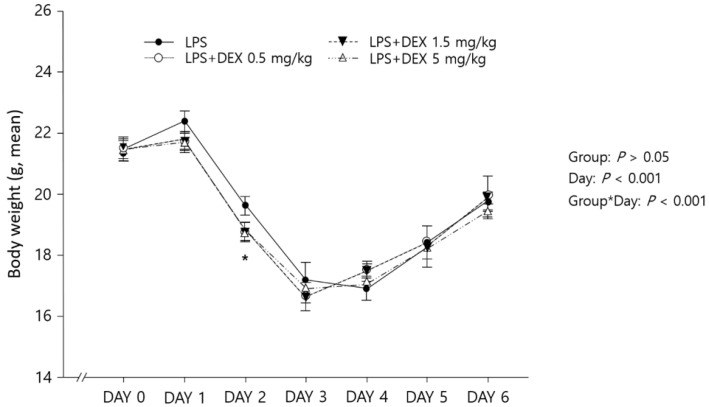
Weight changes in LPS-challenged mice with dexamethasone treatment at different doses (N = 8 per group). Symbols and error bars represent means and standard errors, respectively (* *p* < 0.05, adjusted *p*-value by Bonferroni−Holm method).

Five days after LPS challenge (day 6), the highest survival rate was observed in the mice treated with DEX 5 mg/kg daily (87.5% with LPS + DEX 5 mg/kg vs. 62.5% with LPS + DEX 1.5 mg/kg vs. 62.5% with LPS + DEX 0.5 mg/kg vs. 37.5% in LPS only, *p* < 0.05) (Figure 1). The body weights of the mice significantly changed over time (Figure 2) (*p* < 0.001); however, no significant differences in body weight were observed among the different DEX doses (*p* > 0.05). Specifically, 24 h after LPS injection (day 2), a substantial decline in body weight was observed; the reduction in body weight was greater in DEX-treated groups compared with the LPS-challenged mice without DEX treatment (*p* < 0.001). From day 3, the mice that survived gradually gained weight over time (Figure 2).

### 3.2. Changes in Cytokine Levels with Dexamethasone Administration

**Figure 3:**
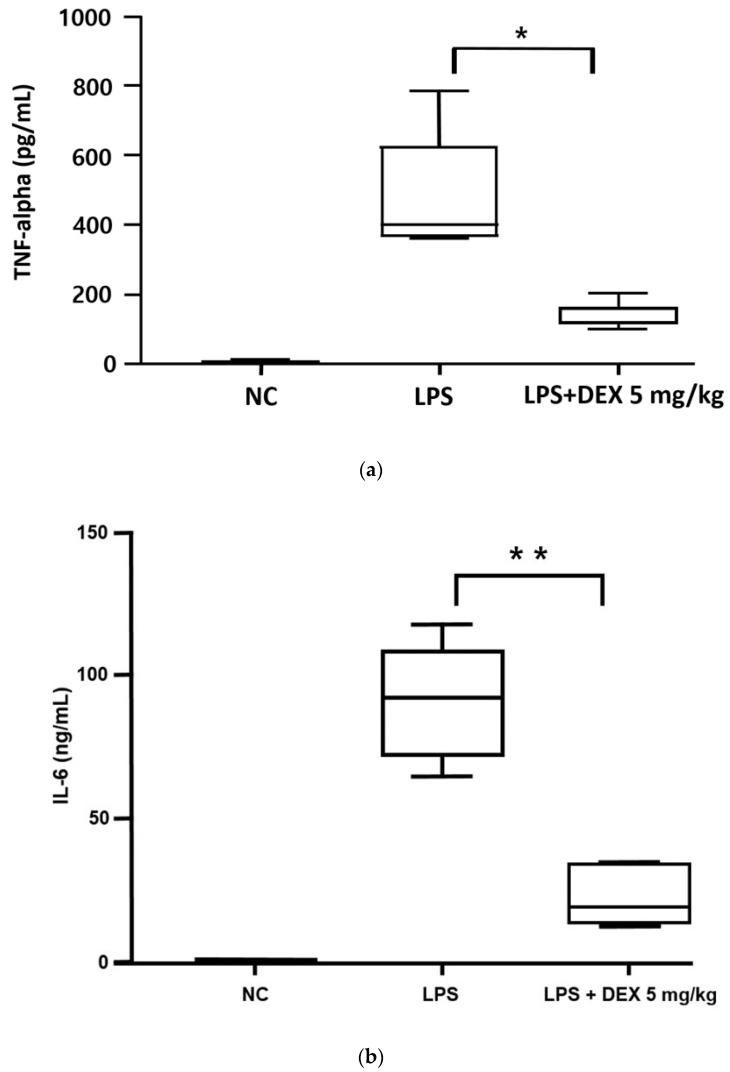
Serum cytokine concentrations 4 h after LPS administration: (**a**) TNF-α and (**b**) IL-6. The data are expressed as mean ± standard error (* *p* < 0.05, ** *p* < 0.01, adjusted *p*-value by Bonferroni-Holm method). NC—normal control.

Compared with the normal control group, the LPS-challenged groups had significantly high serum concentrations of TNF-α (408.83 ± 18.32 pg/mL vs. 5.42 ± 2.35 pg/mL) and IL-6 (91.27 ± 8.56 ng/mL vs. undetectable) (*p* < 0.01 for both). However, with high-dose DEX (5 mg/kg) administered 24 h and 30 min before LPS injection, the increases in the serum concentrations of TNF-α and IL-6 in the LPS-challenged mice were significantly inhibited by 72.03% (TNF-α: 134.41 ± 15.83 pg/mL vs. 408.83 ± 18.32 pg/mL, *p* < 0.05) and 75.81% (IL-6: 91.27 ± 8.56 ng/mL vs. 22.08 ± 4.34 ng/mL, *p* < 0.01), respectively (Figure 3).

## 4. Discussion

To the best of our knowledge, this is the first study evaluating the potential benefits of the early initiation of high-dose oral DEX therapy in sepsis using the LPS-challenged mouse model. We conducted two-series in vivo experiments to explore the effects of high-dose DEX therapy initiated 24 h prior to LPS challenge regarding inflammatory cytokine levels and survival. Consistent with our recent meta-analysis results based on clinical data [8], the survival rate in LPS-challenged mice was significantly improved with early DEX therapy in a dose-dependent manner (Figure 1): higher survival rate for the high-dose DEX group (5 mg/kg of DEX; 87.5%) compared with the lower-dose DEX groups (0.5 mg/kg and 1.5 mg/kg of DEX; 67.5% for both groups). As suggested in previous studies [18,19], significant weight loss occurred in all LPS-challenged mice within 24 h, possibly as a result of the decreased water and food intake (Figure 2). However, compared with the LPS only group, the DEX treatment groups lost significantly more weight, which is consistent with a previous study using a chronic sepsis mouse model [20]. This might be as a result of the potential protective effects of DEX against LPS-induced mortality; the greater survival rate of DEX-treated mice with substantial weight loss after LPS challenge might have contributed to the significantly lower body weight compared with the untreated mice. Although the mechanism for the potential survival benefit of significantly lower body weights in DEX-treated mice after LPS challenge has not been completely elucidated, decreased fluid retention and edema resulting from markedly suppressed levels of cytokines, including TNF-α and IL-6, with the early initiation of corticosteroid therapy may have accounted for the significantly lower body weights and ultimately led to potential survival benefits [1]. Our present study demonstrated significant suppression of serum TNF-α and IL-6 levels in LPS-challenged mice treated with high-dose DEX (5 mg/kg) compared with the untreated controls (Figure 3). In clinical practice, weight loss is rarely observed in patients with sepsis because intensive fluid resuscitation is the mainstay treatment in order to maintain blood pressure [4]. However, according to previous studies, weight gain or edema accompanied by excessive fluid resuscitation or hypoalbuminemia in septic patients significantly increases sepsis-related mortality [21,22]. Future studies evaluating the therapeutic benefits of early, high-dose DEX therapy in patients with sepsis are warranted in order to confirm the clinical utility of early, high-dose glucocorticoid therapy for the management of sepsis.

According to the most recent sepsis guidelines [4], the optimal timing of initiating steroid therapy is unclear; however, in clinical practice, corticosteroids are typically initiated in patients requiring ≥4 h of norepinephrine or epinephrine therapy at a dose of ≥ 0.25 μg/kg/min [4]. In this study, we explored the potential therapeutic effects of early DEX therapy in sepsis by administering the first dose of DEX 24 h prior to LPS challenge. It was found that early initiation led to significant survival benefits as well as to suppression of the excessive inflammatory responses in the LPS-challenged mice (Figure 1 and Figure 3). Sepsis induces hyperactivation of innate immunity in the acute phase, which contributes to early deaths in patients with sepsis through acute, overwhelming inflammation [23,24]. Based on a previous study characterizing the time course of inflammatory cytokines in humans, the maximum increase in the plasma concentrations of TNF-α and IL-6 was observed 2 to 3 h after LPS bolus in the LPS-challenged volunteers [25]. Moreover, a pre-clinical mechanistic study on mortality in early and late sepsis suggested a pertinent association between IL-6 levels and mortality in the early phase of sepsis; the higher the IL-6 levels, the greater the risk of mortality within 4 days of sepsis induction in mice [26]. However, the critical role of increased cytokine levels on mortality is not evident in the chronic phase of sepsis, as suggested by the evidence of death from both immunosuppression and immunostimulation in mice, implying the importance of the early suppression of cytokine levels for preventing early sepsis-related mortality [26]. Corticosteroids, including DEX, suppress the cells involved in innate immunity and thus prevent the excessive production and release of pro-inflammatory cytokines such as TNF-α and IL-6 (Figure 3). This might account for the survival benefits of early DEX therapy in LPS-challenged mice, as shown in our current study (Figure 1). Our findings are consistent with those of a previous study that demonstrated a significantly lower in-hospital mortality in sepsis patients already on immunosuppressive drugs, including corticosteroids, at the time of sepsis diagnosis compared with those who were not [27]. These results highlight the importance of rapid and accurate diagnosis of sepsis for the early initiation of corticosteroid therapy in order to improve treatment outcomes. The delayed initiation of corticosteroids (i.e., ≥4 to 6 h) after recognizing sepsis in previous studies might account for the reported harmful effects or lack of benefits associated with corticosteroid therapy [28]. However, rapid, accurate diagnosis of sepsis, especially in critically ill patients, is challenging because of the presence of inflammation associated with underlying non-infectious diseases, as well as slow confirmation of infection based on microbiological cultures [29]. Therefore, further research is warranted for the rapid, accurate diagnosis of sepsis in order to maximize the potential therapeutic benefits of early corticosteroid therapy.

Although the optimal dose and relative glucocorticoid-to-mineralocorticoid activity of corticosteroid therapy for sepsis are still obscure, the most recent guidelines suggest intravenous hydrocortisone with a relatively high mineralocorticoid activity at low doses (e.g., 200 mg daily) in order to maintain blood pressure in vasopressor-dependent patients with septic shock [4]. This highlights the importance of mineralocorticoid effects for the management of septic shock via accelerating hemodynamic stabilization [7]. In contrast, our present study indicated the potential therapeutic benefits of high glucocorticoid activity during the early phase of sepsis (Figure 1 and Figure 3). The protective effects of DEX on mortality appeared to be dose-dependent, with the lowest mortality observed in the highest DEX dose group among LPS-challenged mice (Figure 1). However, previous clinical studies in patients with sepsis or septic shock suggested no therapeutic benefits, or even potential harm, as a result of high-dose glucocorticoid therapy [30]. Favorable outcomes of high-dose glucocorticoid therapy in our present study might result from the early initiation of DEX evaluated in LPS-challenged mice; in previous studies, high-dose glucocorticoids were administered at least 4 to 6 h after a documented, definitive diagnosis of sepsis in patients. In fact, other previous clinical studies have suggested the potential survival benefits of high-intensity glucocorticoid therapy when administered early in the course of sepsis [31]. Nonetheless, authentic evaluation on the administration time and dose of DEX is warranted based on recent study results demonstrating that prophylactic intraperitoneal administration of excessively high-dose DEX (10 mg/kg i.p. every 12 h) 3 days prior to LPS injection (20 mg/kg) resulted in the death of all mice within 24 h of LPS challenge, indicating that the excessive suppression of pro-inflammatory effects by extensively high-dose DEX contributes to a substantially worse prognosis [32]. The pathophysiological nature of sepsis is quite complicated, secondary to fluctuating immune function during the courses of sepsis progression [33]. Studies have suggested that sepsis induces a pro-inflammatory response, resulting in systemic inflammatory response syndrome, subsequently inducing organ failure in the early phase, as well as compensatory anti-inflammatory responses, inducing immunosuppression and late death, and substantially increased re-positive blood cultures associated with opportunistic infection in the later phase of sepsis, secondary to sepsis-induced immunosuppression. This further accentuates the imperative aspects of fluctuating immune function in sepsis progression and management [1,33,34]. Furthermore, the LPS-challenged mouse model used in our current study may have only mimicked the acute excessive inflammatory response to sepsis or a certain subset of patients with sepsis or septic shock, called the “inflammopathic” subtype, both of which are associated with hyper-activation of the innate immune system and a higher mortality rate [24]. Conflicting clinical evidence regarding the therapeutic effects of high-intensity glucocorticoids in sepsis might result from the substantial heterogeneity in the type and severity of sepsis, depending on the host molecular patterns implicated in the pathogenesis [24]. Therapeutic benefits of high-dose glucocorticoids, such as high-dose DEX, might be exclusively limited to a certain subset of patients, including those with the “inflammopathic” subtype of sepsis. Therefore, future studies evaluating the dose-dependent clinical outcomes of DEX therapy, in relation to fluctuating immune function during sepsis prognosis to determine the exact dose and time of DEX administration in sepsis patients are needed. Moreover, identifying the biomarkers predictive of the responses to steroid therapy in sepsis will promote the practice of precision medicine for the management of sepsis and/or septic shock.

Traditional sepsis management involves antibiotic treatment followed by fluid resuscitation, and the initiation of corticosteroid is recommended in patients requiring ≥4 h of norepinephrine or epinephrine therapy at a dose of ≥0.25 μg/kg/min [4]. However, some clinicians advocate for the early initiation of corticosteroids with antibiotics, fluid resuscitation, and vasopressors in septic shock patients [35]. This study provides pivotal evidence on the early initiation of DEX in sepsis patients, because this is the first study that demonstrates the beneficial prognosis of the early initiation of oral DEX therapy in LPS-challenged mice, implying the significant importance of DEX-induced early anti-inflammatory activities in sepsis progression. Previously, several studies have investigated the various effects of DEX in LPS-challenged mice [32,36,37,38]. However, the administration times and routes were considerably different from this study; these studies administered DEX intraperitoneally, one of the most common administration sites in mice [39], 1 h prior to LPS injection. Dexamethasone exhibits dose-dependent pharmacokinetic profiles reaching a maximal plasma level within 1.6 and 2.0 h after administration [40]; however, the onset of action is usually delayed by 3 to 8 h [41]. Thus, DEX administration 1 h prior to LPS injection, which significantly increases the serum cytokines within 2–3 h, secondary to the immediate induction of inflammatory activities, may not be sufficient to promote the early suppression of pro-inflammatory hyperactivation in LPS-challenged mice as we hypothesized. Nonetheless, the current study design still has limitations, as early corticosteroid including DEX may be impractical in real clinical settings, as the early diagnosis of sepsis is challenging in most occasions, subsequently also delaying antibiotic administration [42,43]. Furthermore, obscurity on the impact of DEX doses on the degree of immunosuppression still exists. Hence, future studies evaluating clinical significance of immunosuppression, notwithstanding absence of mineralocorticoid activity in patients with sepsis and septic shock, are warranted in order to advocate for early DEX use in clinical practice.

Considering the exploratory nature of this study, caution needs to be exercised when interpreting our current study findings. Although the LPS-challenged animal model is considered to be the most suitable for investigating acute, systemic inflammation [17], it does not exactly reproduce the pathophysiologic processes of human sepsis, as it has earlier and greater cytokine responses as well as a shorter duration than in humans. In addition, our present study evaluated the potential therapeutic benefits of corticosteroid therapy in LPS-injected mice only using dexamethasone. Considering the substantial variability in the relative glucocorticoid-to-mineralocorticoid activity among different corticosteroids, additional corticosteroids with variable glucocorticoid and mineralocorticoid activities should be comparatively evaluated for their potential therapeutic benefits in sepsis and septic shock. Moreover, although this study revealed a pertinent association between the substantially elevated survival rate and a reduction in cytokine levels with the high-dose DEX (5 mg/kg) treated group, additional cytokine analyses in low-dose DEX (0.5 and 1.5 mg/kg) groups would have provided more comprehensive results regarding the impact of DEX on cytokine suppression and improved mortality. Therefore, our study findings should not be generalized to all corticosteroid therapy initiated at different times throughout the disease course in real-world patients with sepsis. Large-scale, clinical studies, preferably with in vitro mechanistic assessment, using various corticosteroids administered at different timing to confirm therapeutic roles, as well as optimal regimens of corticosteroids in sepsis, are imperative.

## 5. Conclusions

In conclusion, early initiation of DEX therapy significantly improved survival in LPS-challenged mice. The survival benefit of DEX was greater at a higher dose (i.e., 5 mg/kg) compared with lower doses (i.e., 0.5 and 1.5 mg/kg). High-dose DEX therapy significantly reduced the serum concentrations of pro-inflammatory cytokines, specifically TNF-α and IL-6, in the LPS-injected mice. Overall, our current exploratory study suggests early initiation of DEX as a promising adjunctive therapy for sepsis through prompt suppression of acute excessive inflammation. These study results may provide pivotal evidence for improving early clinical outcomes in sepsis patients through the early initiation of DEX, implied by increased survival rates associated with enhanced quality of patient care. However, cautious interpretation is required regarding the dose and time of DEX administration as the mouse model does not exhibit the exact pathophysiology of sepsis in humans. Thus, further studies on clinical prognosis associated with corticosteroid therapy, in terms of fluctuating aspects of immune function during sepsis progression, as well as identification of the predictive biomarkers related to responses to steroid therapy in sepsis, are warranted in order to promote precision management of sepsis.

## Data Availability

Not applicable.
